# The necroptosis signature and molecular mechanism of lung squamous cell carcinoma

**DOI:** 10.18632/aging.205210

**Published:** 2023-11-15

**Authors:** Guo-Qiang Song, Hua-Man Wu, Ke-Jie Ji, Tian-Li He, Yi-Meng Duan, Jia-Wen Zhang, Guo-Qiang Hu

**Affiliations:** 1 Department of Pulmonary, Changxing County Hospital of Traditional Chinese Medicine, Huzhou, China; 2 Department of Pulmonary and Critical Care Medicine, Zigong First People’s Hospital, Zigong, China; 3 Department of Radiotherapy, Changxing People’s Hospital, Huzhou, China; 4 Department of Cancer Center, Changxing County Hospital of Traditional Chinese Medicine, Huzhou, China

**Keywords:** lung squamous cell carcinoma (LUSC), necroptosis, TCGA datasets, GEO datasets, bioinformatics analysis

## Abstract

Background: Given the poor prognosis of lung squamous cell carcinoma (LUSC), the aim of this study was to screen for new prognostic biomarkers.

Methods: The TGCA_LUSC dataset was used as the training set, and GSE73403 was used as the validation set. The genes involved in necroptosis-related pathways were acquired from the KEGG database, and the differential genes between the LUSC and normal samples were identified using the GSEA. A necroptosis signature was constructed by survival analysis, and its correlation with patient prognosis and clinical features was evaluated. The molecular characteristics and drug response associated with the necroptosis signature were also identified. The drug candidates were then validated at the cellular level.

Results: The TCGA_LUSC dataset included 51 normal samples and 502 LUSC samples. The GSE73403 dataset included 69 samples. 159 genes involved in necroptosis pathways were acquired from the KEGG database, of which most showed significant differences between two groups in terms of genomic, transcriptional and methylation alterations. In particular, CHMP4C, IL1B, JAK1, PYGB and TNFRSF10B were significantly associated with the survival (*p* < 0.05) and were used to construct the necroptosis signature, which showed significant correlation with patient prognosis and clinical features in univariate and multivariate analyses (*p* < 0.05). Furthermore, CHMP4C, IL1B, JAK1 and PYGB were identified as potential targets of trametinib, selumetinib, SCH772984, PD 325901 and dasatinib. Finally, knockdown of these genes in LUSC cells increased chemosensitivity to those drugs.

Conclusion: We identified a necroptosis signature in LUSC that can predict prognosis and identify patients who can benefit from targeted therapies.

## INTRODUCTION

The overall incidence of non-small cell lung cancer (NSCLC) was 40.9 per 100,000 in 2017, and that for age groups <65 years and ≥65 years were 13.5/100,00 and 230/100,00 respectively [1]. Squamous cell carcinoma of the lung (LUSC), a type of NSCLC, is currently the second most commonly diagnosed cancer worldwide with an incidence rate of about 30% [1]. Although recent advances in immunotherapy have prolonged the survival of LUSC patients [2], studies show that patients in the advanced stage do not benefit from PD-1 or PD-L1 checkpoint blockers [3]. Therefore, it is essential to identify novel therapeutic targets for LUSC.

Necroptosis is a form of programmed inflammatory cell death that involves death domain receptors [4], and is frequently dysregulated in many inflammatory diseases [5]. Recent studies have shown that necroptosis plays a key role in tumorigenesis and metastasis, and can be targeted as a novel anti-tumor strategy [6, 7]. Although the molecular mechanisms of necroptosis have been largely elucidated, relatively little is known regarding its regulation and function in tumor cells [8, 9]. In addition, the exact role of necrosis in tumor development remains controversial [10]. Nevertheless, necroptosis has gained attention as a potential therapeutic target in NSCLC and small cell lung cancer (SCLC) [11, 12]. For instance, RIPK1, RIPK3 and MLKL, the key regulators of necroptosis, are downregulated in NSCLC and correlated to prognosis [13]. In addition, 7 necroptosis-related long non-coding RNAs (lncRNAs), including AC026355.2, AC099850.3, AF131215.5, UST-AS2, ARHGAP26-AS1, FAM83A-AS1 and AC010999.2, can predict the prognosis for lung adenocarcinoma (LUAD) patients [14]. A recent study showed that necroptosis-related genes (NRGs) are strongly associated with tumor mutational burden (TMB), tumor immune microenvironment and prognosis [15]. Furthermore, low expression levels of the necroptosis markers RIPK3 and PELI1 are associated with increased mortality in the squamous cell carcinoma subtype of NSCLC [16].

The aim of this study was to evaluate the relationship between a necroptosis-related signature and clinical outcomes in LUSC patients, and explore the potential molecular mechanisms and drug responses associated with necroptosis.

## MATERIALS AND METHODS

### Data download and preprocessing

The expression profile, clinical information and survival data of LUSC patients were downloaded from the UCSC Xena database (https://xenabrowser.net/datapages/), and this TCGA_LUSC dataset was used as the training set. The gene expression data were downloaded in the log2(norm_count+1) format, converted into TPM value and log2 transformed for subsequent analysis. The GSE73403 [17] dataset was downloaded from the Gene Expression Omnibus (GEO) database as the validation set (Table 1). The genes related to necroptosis pathways were downloaded from the KEGG database (https://www.genome.jp/kegg/) using “map04217” as the search item.

**Table 1:** Datasets.

**Dataset ID**	**GPL**	**Control**	**Tumor**	**Note**
TCGA-LUSC	/	51	502	Training set
GSE73403	GPL6480	/	69	Validation set

### Definition of necroptosis signature

The GSEA function of the “clusterProfiler” package was used to analyze the differences in necroptosis pathways between LUSC and control groups, and between the different LUSC subgroups based on age (≥60 and <60) and tumor stage (stage I + II vs. stage III + IV). Based on the transcriptomic data of the training set and KEGG necroptosis pathways, the ssGESA algorithm in the “GSVA” package was used to calculate the sample enrichment score (parameters were kcdf = “Gaussian”, abs.ranking = F).

### Screening of altered necroptosis genes

The mutation and copy number variation (CNV) data of LUSC were downloaded from the TCGA database. The “matfoos” package was used to display the overall mutation and CNV status of necroptosis genes, and the “Rcircos” package was used to draw their chromosomal distribution map. Based on TCGA RNA-seq data, the differentially expressed necroptosis genes between LUSC and normal lung samples were screened using the “limma” package, and the differences in the expression levels of the necroptosis genes between different clinical subgroups (immunotype, stage, smoking history, ALK-eml4 rearrangement, age, gender, etc.) were analyzed by Wilcox test. The patients in TCGA_LUSC cohort were divided into the respective low- and high-expression groups based on the median expression level of each necroptosis gene, and the differences in overall survival (OS) were analyzed by the Kaplan-Meier method using the “survival” package. The methylation data of LUSC were downloaded from the TCGA database, and the differential methylation levels of necroptosis genes between LUSC and normal lung samples were calculated using the “ChAMP” package.

### Correlation of necroptosis signature with prognosis and clinical characteristics

The patients in the training set were divided into the high- and low-risk groups using the surv_cutpoint function to find the optimal grouping threshold, and the OS of the groups was compared. The prognostic significance of the necroptosis signature was verified in the GSE73403 dataset. Univariate and multivariate Cox regression analyses were performed to evaluate the correlation between the necroptosis signature and clinical characteristics in the training and validation sets. The impact of the signature on the outcomes of immunotherapy was evaluated on the basis of immunotherapy data from bladder cancer (IMvigor210) [18], melanoma GSE91061 [19] and renal clear cell carcinoma (PMID: 32472114) datasets [20].

### Correlation of the necroptosis signature with molecular features, immune infiltration, and drug response

Based on the CNV data from TCGA, the “matfoos” package was used to display the waterfall plot of the mutation rate of necroptosis genes. Fisher’s exact test was used to identify the differences in the CNVs between the two risk groups. The KEGG pathway gene collection “c2.cp.kegg.v7.4.symbols.gmt” was downloaded from the MsigDB database (http://www.gsea-msigdb.org/), and the pathway enrichment scores were calculated based on ssGSEA. Differential pathways between the two groups were screened using the “limma” package. The TIMER database (https://cistrome.shinyapps.io/timer/), and the CIBERSORT, XCELL and TIMER algorithms were used to calculate the immune cell infiltration in LUSC samples. In addition, the necroptosis signature was also calculated based on the GDSC (https://www.cancerrxgene.org/) and CCLE (https://sites.broadinstitute.org/ccle/) databases. The correlation between the signature and drug sensitivity (IC50) was calculated by Spearman method, and the differences between the risk groups were compared.

### Validation of drug sensitivity at the cellular level

The A549 cells (ATCC, USA) were transfected with siRNAs targeting specific genes and the scrambled controls (Sigma-Aldrich, USA) using Lipofectamine 2000 (Invitrogen, USA) according to the manufacturer’s instructions (Table 2). IL-1β siRNA was designed by GenePharma (China) and transfected using Lipofectamine™ 2000 (11668030, Thermo Fisher, USA). Briefly, the cells were incubated with 10 μl siRNA and 5 μl Lipofectamine 2000 in DMEM (total volume 500 μl/well) for 24 hours. The medium was discarded and fresh DMEM supplemented with 10% FBS was added (1 ml/well), followed by incubation for additional 48 hours. Total RNA was extracted from the transfected cells using Trizol Reagent (Invitrogen, USA), and reverse transcribed using RT reagent Kit gDNA Eraser (TaKaRa, Japan). QRT-PCR was performed using SYBR-Green Master Mix (TaKaRa) with GAPDH as the internal reference (Table 3). Each sample was analyzed in triplicates. The relative expression levels of the genes were calculated using the 2^−ΔΔCt^ method. The experiment was repeated thrice.

**Table 2:** Sequences of siRNAs.

**siRNA**	**Forward (F): Sequence**	**Reverse (R): Sequence**	**Target gene**
siIL-1β#1	5′-GGUGAUGUCUGGUCCAUAUTT-3′	5′-AUAUGGACCAGACAUCACCTT-3′	Human IL1β
siIL-1β#2	5′-GCGUGUUGAAAGAUGAUAATT-3′	5′-UUAUCAUCUUUCAACAUGCTT-3′	Human IL1β
siCHMP4C#1	5′-CACUCAGAUUGAUGGCACA-3′		Human CHMP4C
siCHMP4C#2	5′-CCUGCGUCUCUACAACUA U-3′		Human CHMP4C
siJAK1	5′-GCCUGAGAGUGGAGGUAAC-3′	5′-GUUACCUCCACUCUCAGGC-3′	Human JAK1
siPYGB#1	5′-GGUCCUGUAUCCAAAUGAU-3′		Human PYGB
siPYGB#2	5′-CCCUGUACAAUCGAAUCAA-3′		Human PYGB
siNC	5′-CCUCUGGCAUUAGAAUUAUTT-3′		Negative control

**Table 3:** Primer sequences for target genes.

**Gene**	**Forward (F):**	**Reverse (R):**	**Target gene**
hIL-1β	5′-TGATGGCTTATTACAGTGGCA-3′	5′-GGTCGGAGATTCGTAGCTGG-3′	Human IL-1β
hCHMP4C	5′-AGACTGAGGAGATGCTGGGCAA-3′	5′-TAGTGCCTGTAATGCAGCTCGC-3′	Human CHMP4C
hJAK1	5′-GTCCCTGAAGCCTGAGAGTG-3′	5′-CTTGATACCATTGCCTCCGT-3′	Human JAK1
hPYGB	5′-ACGCAGCAGCACTACTAC-3′	5′-TCGCAGGCATTCTGAAGG-3′	Human PYGB
hGAPDH	5′-TGTGTCCGTCGTGGATCTGA-3′	5′-CCTGCTTCACCACCTTCTTGA-3′	Negative control

Trametinib (S2673), dasatinib (S1021), PD0325901 and selumetinib (AZD6244) were purchased from Selleck Chemicals (USA). SCH772984 was purchased from Ablome (USA). The drugs were dissolved in dimethyl sulfoxide (DMSO) to yield 5 or 10 mM stock solutions and stored at −80°C. The transfected cells were harvested during logarithmic growth phase and seeded in 96-well plates at the density of 200 cells/well in a final volume of 190 μL/well. After 24 hours, 10 μL of the respective drugs (25 nM trametinib, 1 nM dasatinib, 0–1 mM PD0325901, 2 μM SCH772984 group, and 10 nM selumetinib) was added, and the cells were cultured for 144 hours.

### Statistical analysis

All statistical analyses were performed using R 4.1.1 (http://www.Rproject.org), GraphPad Prism 7 software, and ImageJ software. A statistically significant threshold was considered when *p* < 0.05. The *t*-test was employed to evaluate normally distributed data, while the Mann-Whitney *U* test was utilized for assessing non-normally distributed data. The overall survival was analyzed by the Kaplan-Meier method in different groups, and univariate and multivariate Cox regression analyses were performed to evaluate the correlation between the necroptosis signature and clinical characteristics in the training and validation sets.

The correlation between the signature and drug sensitivity (IC50) was calculated by Spearman method.

## RESULTS

### Data download and preprocessing

The TCGA_LUSC training set included 51 control samples and 502 tumor samples. GSE73403 included 69 tumor samples as a validation set. In addition, 159 genes involved in necroptosis pathways (map04217) were obtained from the KEGG database.

### Definition of necroptosis signature

**Figure 1:**
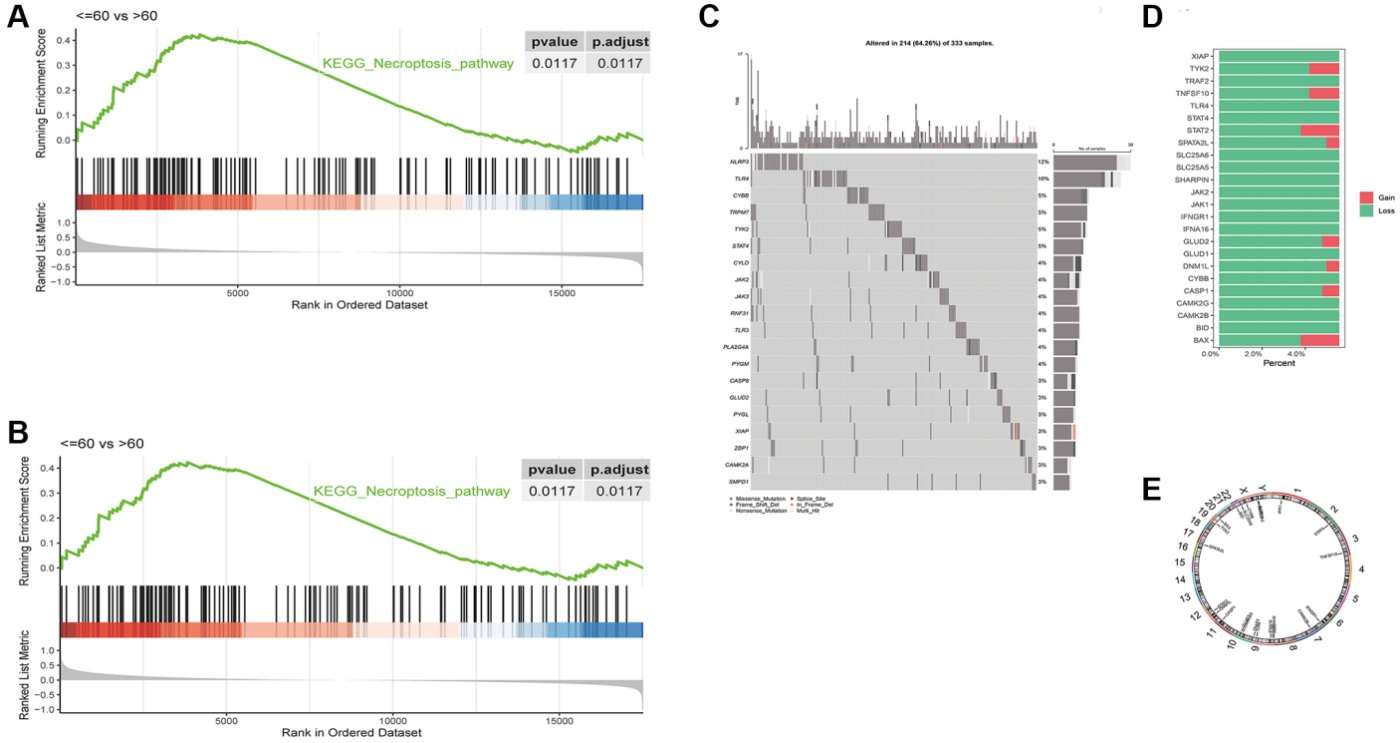
Differences in GSEA enrichment scores of necroptosis pathway between in LUSC subgroups based on (**A**) age and (**B**) tumor stage. (**C**) Waterfall plot of the top 20 necroptosis genes with the highest mutation rates. (**D**) CNV distribution of 24 necroptosis genes, GAIN - amplification and LOSS - deletion. (**E**) Chromosomal distribution of 24 necroptosis genes.

The necroptosis pathways showed significant differences in enrichment between the different age groups of LUSC patients (*p* = 0.0117, Figure 1A), but not between the different stages (Figure 1B). The enrichment score file for each sample in TCGA-LUSC can be found in: (Supplementary Table 1).

### The necroptosis-related genes were significantly altered in the tumors

We analyzed the mutation data of 124 necroptosis genes, and the waterfall plot of the top 20 necroptosis genes with the highest mutation rates is shown in Figure 1C. The CNV data was available for 24 genes, and as shown in Figure 1D, most genes showed loss of function. The chromosomal distribution of these 24 necroptosis genes is shown in Figure 1E, and the genes mapped to chromosomes 1, 2, 3, 6, 7, 8, 9, 10, 11, 12, 17, 19, 22, X and Y.

**Figure 2:**
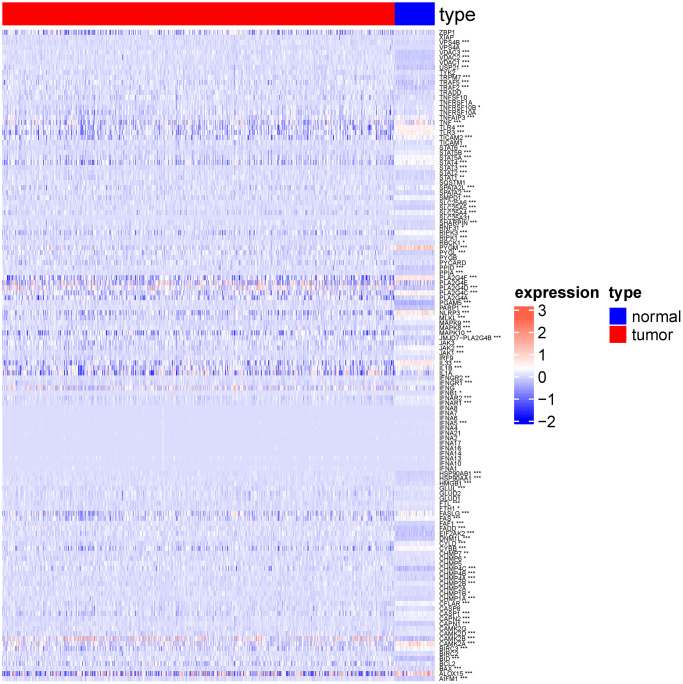
**Heat map showing expression of necroptosis genes in normal group and lung squamous cell carcinoma group.** (^*^p < 0.05; ^**^p < 0.01; ^***^p < 0.001; ^****^p < 0.0001).

**Figure 3:**
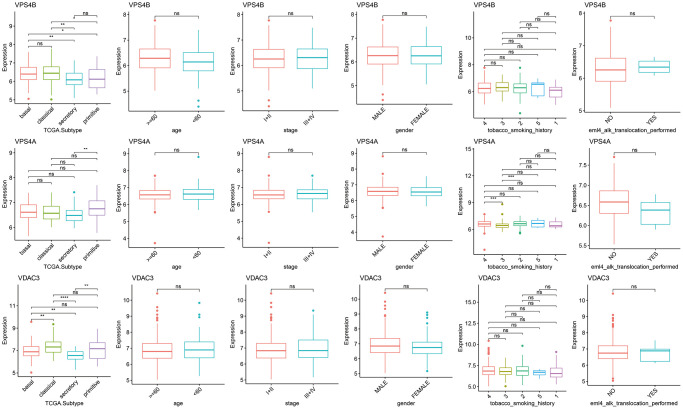
**Differences in the expression of necroptosis genes among the clinical subgroups.** (^*^p < 0.05; ^**^p < 0.01; ^***^p < 0.001; ^****^p < 0.0001; ns *p* > 0.05).

As shown in the heat map in Figure 2, most of 130 necroptosis genes were differentially expressed between the LUSC and normal lung samples of TCGA-LUAD cohort, and the differences were statistically significant (*p* < 0.05). We analyzed the expression profiles of 3 random necroptosis genes in the different clinical subgroups. As shown in Figure 3, VPS4B and VPS4A showed differential expression in the patients with smoking history, VDAC3 was only differentially expressed in the TCGA. Subtype Expression (*p* < 0.05, Figure 3).

**Figure 4:**
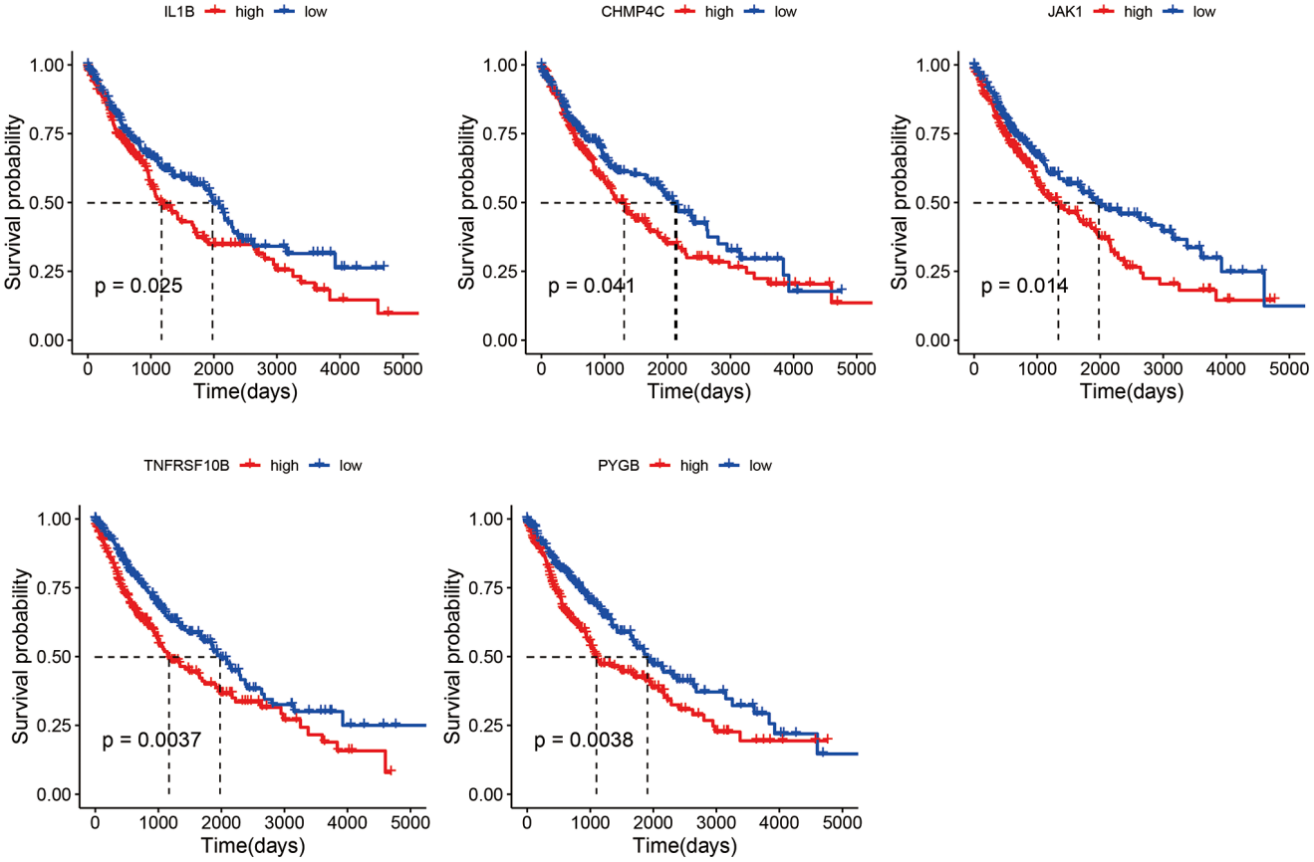
Survival curves of LUSC patients demarcated on the basis of the median expression of necroptosis genes.

Among the differentially expressed necroptosis genes in the TCGA-LUSC dataset, only CHMP4C, IL1B, JAK1, PYGB and TNFRSF10B were significantly associated with the OS (Figure 4). The patients were divided into the respective high- and low-expression groups based on the median expression of each gene.

**Figure 5:**
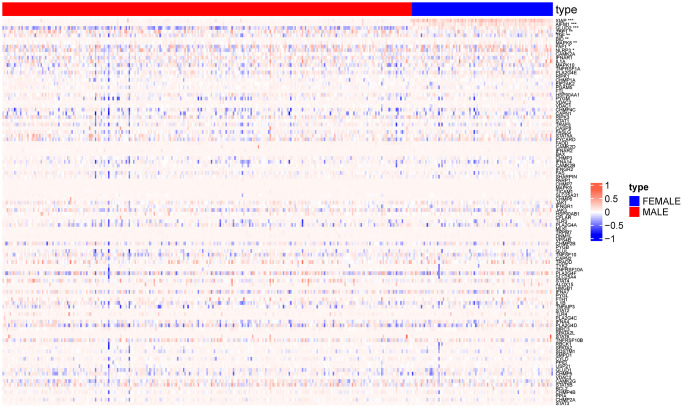
**Heat map showing methylation changes in necroptosis genes in male and female patients.** (^*^p < 0.05; ^**^p < 0.01; ^***^p < 0.001).

The methylation data of 104 necroptosis genes were also analyzed, we revealed significant differences in the methylation status of a few genes among the male and female patients in TCGA-LUSC cohort (*p* < 0.05, Figure 5).

### The necroptosis signature is associated with the prognosis and clinical characteristics of LUSC

**Figure 6:**
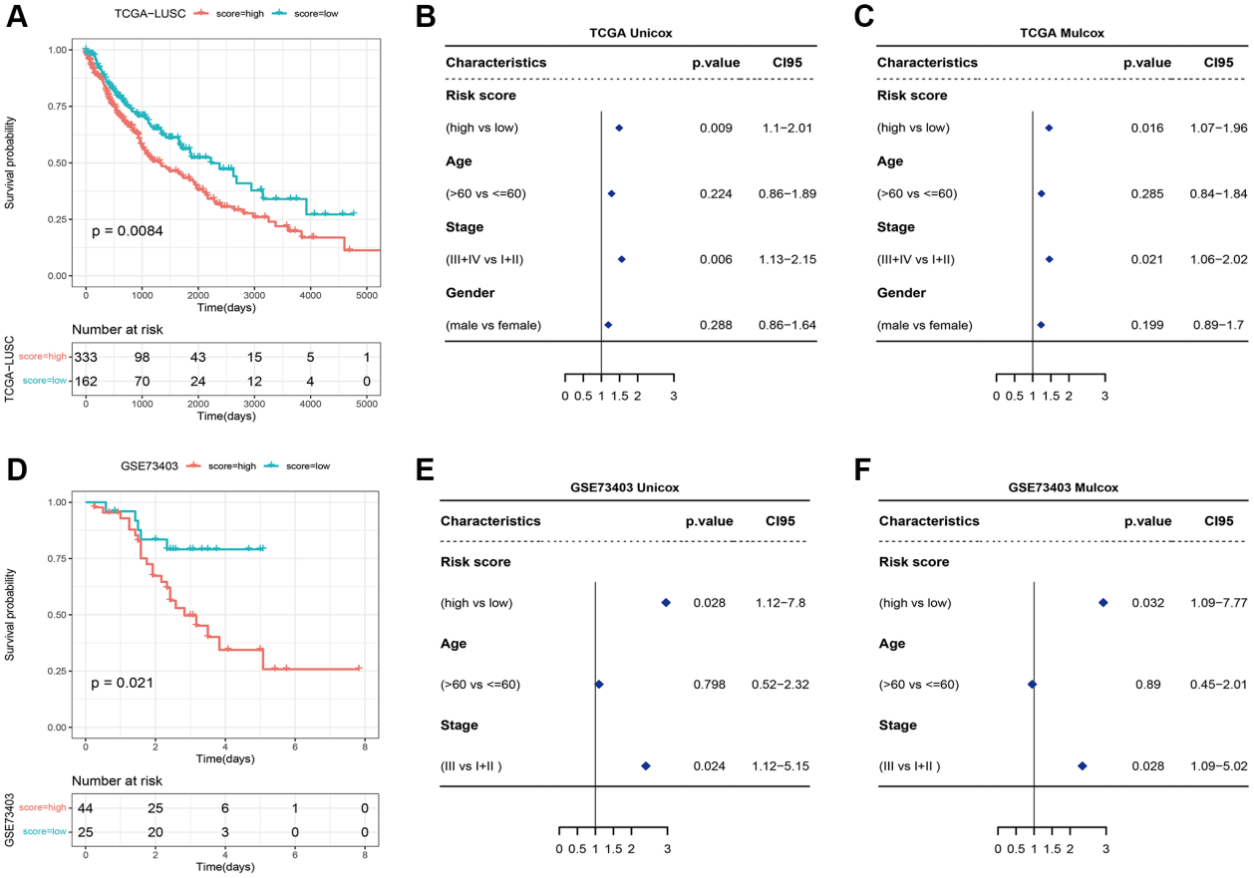
(**A**) Survival curves of high-risk and low-risk groups based on the necroptosis signature. (**B**) Univariate and (**C**) multivariate analyses of the necroptosis signature. (**D**) Survival curve of high-risk and low-risk groups in the GSE73403 dataset. (**E**) Univariate and (**F**) multivariate Cox analyses of the necroptosis signature in the GSE73403 dataset.

Based on the OS data of TCGA_LUSC, the patients were divided into the high- and low-risk groups using the optimal grouping threshold of 2.005388 as calculated by surv_cutpoint function. As shown in Figure 6A, the high-risk patients had worse survival compared to the patients in the low-risk group (*p* = 0.0084), indicating that the necroptosis signature has prognostic significance. Furthermore, the necroptosis signature was identified as an independent prognostic factor for LUSC according to the univariate (*p* = 0.009) and multivariate (*p* = 0.016) Cox analyses (Figure 6B, 6C).

**Figure 7:**
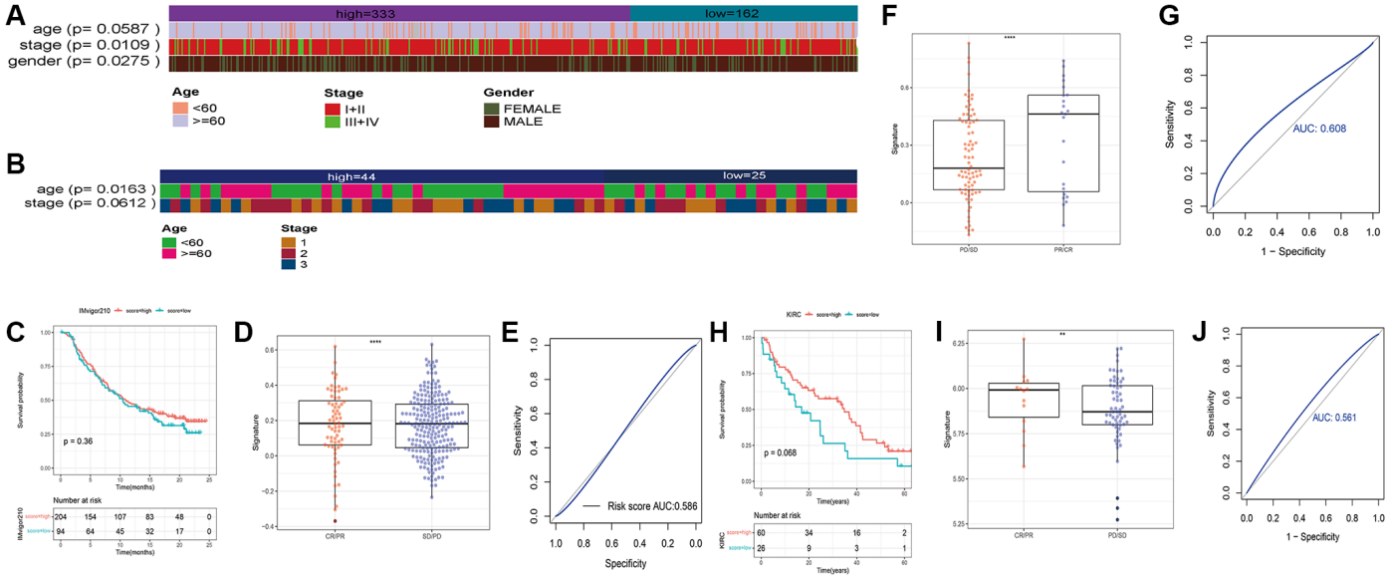
Heatmaps showing of the distribution of clinical features between the two risk groups in (**A**) TCGA_LUSC and (**B**) GSE73403 datasets. (**C**) Survival of bladder cancer patients grouped on the basis of necroptosis signature (IMvigor210). (**D**) Treatment response of the high-risk and low-risk groups (^***^p < 0.0001). (**E**) ROC curve showing predictive capacity of the necroptosis signature for treatment response; (**F**) Difference analysis of treatment response of the high-risk and low-risk groups in GSE91061 (^****^p < 0.0001). (**G**) ROC curve showing predictive capacity of the necroptosis signature for treatment response. (**H**) Survival of renal clear cell carcinoma patients grouped on the basis of necroptosis signature. (**I**) Treatment response of the high-risk and low-risk groups (^**^p < 0.01). (**J**) ROC curve showing predictive capacity of the necroptosis signature for treatment response.

We also verified the signature in the external dataset GSE73403 using the optimal grouping threshold of 1.213578. As shown in Figure 6D, the high-risk group had worse survival compared to the low-risk group (*p* = 0.021). In addition, the necroptosis signature was validated as an independent prognostic factor by univariate (*p* = 0.028) and multivariate (*p* = 0.032) Cox analyses (Figure 6E, 6F) as in the training set. As shown in Figure 7A, 7B, the distribution of tumor stages and gender were significantly different between the two risk groups in the TCGA_LUSC dataset (*p* < 0.05), whereas age was correlated with the risk score in the GSE73403 dataset (*p* = 0.0163).

To further explore the correlation between the necroptosis signature and the response to immunotherapy, we evaluated the prognosis of patients in independent cancer cohorts including immunotherapy data. As shown in Figure 7C–7E), the necroptosis signature was not significantly correlated with the survival of bladder cancer patients in the IMvigor210 dataset (Figure 7C), but there was a significant difference in the response of the high-risk and low-risk patients after immunotherapy (*p* < 0.0001, Figure 7D). The prediction rate for patient response was 58.6% (Figure 7E). In the GSE91061 dataset of melanoma patients, the necroptosis signature was associated with the response to immunotherapy (*p* < 0.0001, Figure 7F), and the prediction rate was 60.8% (Figure 7G). Unfortunately, the survival data was not available for these patients. Furthermore, there was no difference in the survival prognosis of the low-risk and high-risk clear cell renal cell carcinoma patients (Figure 7H), while the response to immunotherapy showed significant differences (*p* < 0.0001, Figure 7I). The prediction rate of patient response was 56.1% (Figure 7J).

### Analysis of necroptosis signature-related molecular features and drug response

**Figure 8:**
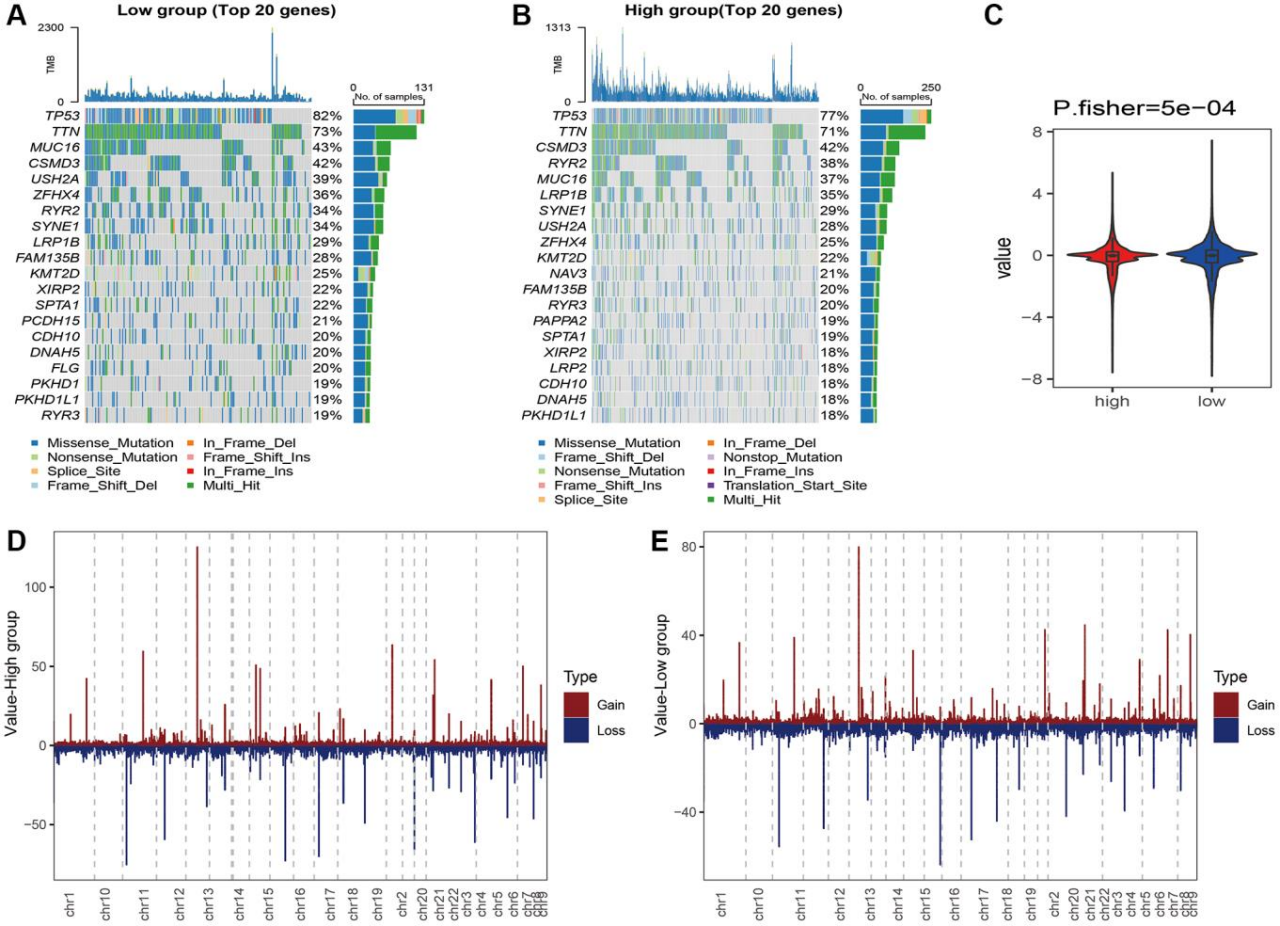
Waterfall plot of the top 20 genes with the highest mutation rates in the (**A**) high-risk and (**B**) low-risk groups. (**C**) CNVs of the necroptosis gene in the risk groups. Distribution of CNVs in the (**D**) high-risk and (**E**) low-risk groups.

**Figure 9:**
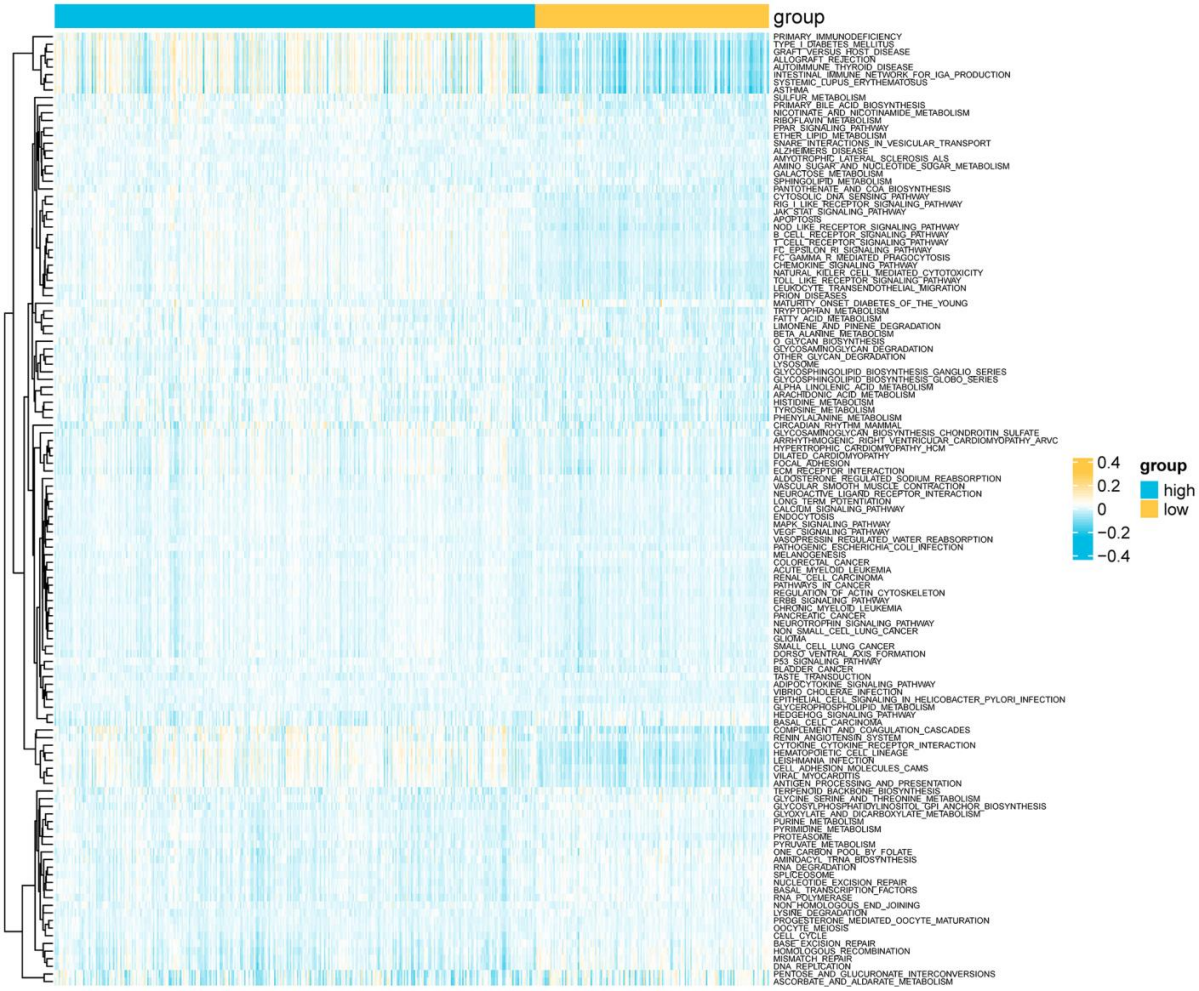
Heatmap showing differential KEGG pathways between the risk groups according to ssGSEA.

We analyzed the genomic alterations between the risk groups. The top 20 genes with the highest mutation rates in the two groups are shown in Figure 8A–8C). Furthermore, the CNVs of the necroptosis genes were significantly higher in the high-risk group compared to that in the low-risk group (*p* = 5e-04, Figure 8D, 8E). A total of 186 KEGG pathways were downloaded from the MsigDB database, and their enrichment scores in the risk groups were evaluated by the ssGSEA algorithm (Supplementary Table 2). As shown in Figure 9, there were 125 significantly different pathways (adj *P* value < 0.01) between the two groups.

**Figure 10:**
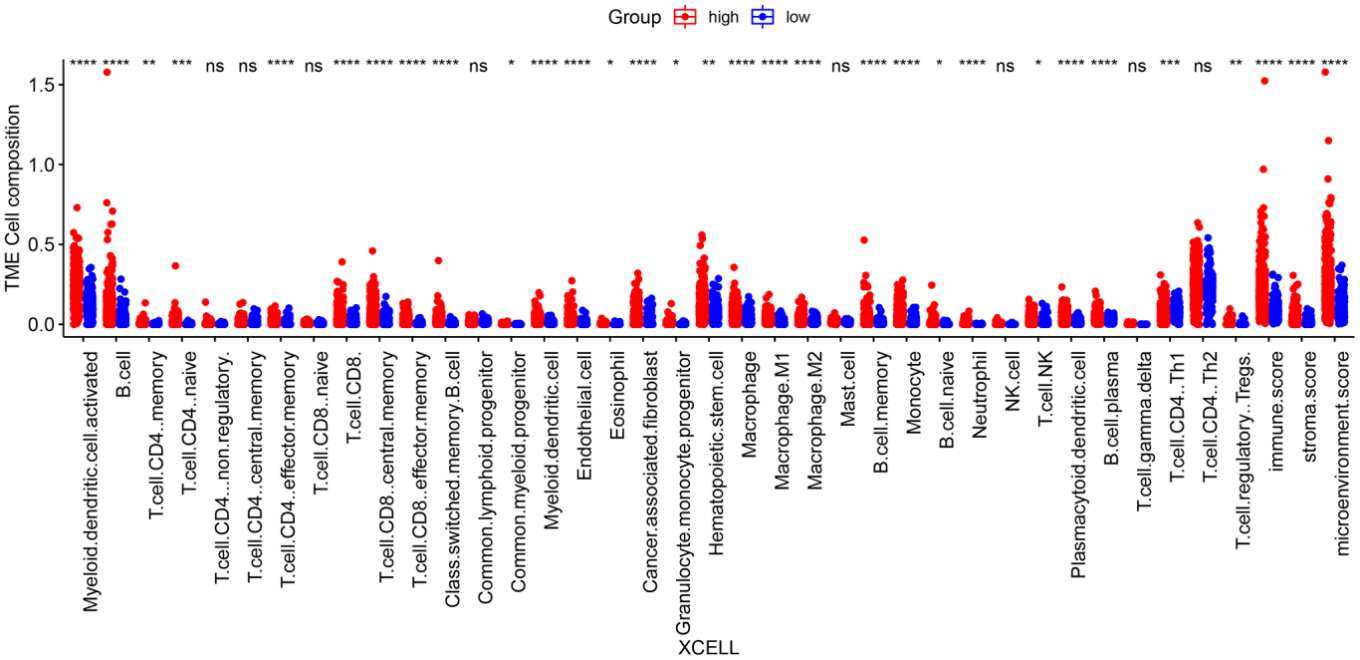
**Differences in immune cell infiltration between high-risk and low-risk groups according to the XCELL algorithm**. (^*^p < 0.05; ^**^p < 0.01; ^***^p < 0.001; ^****^p < 0.0001; ns *p* > 0.05).

We next evaluated the differences in the immune landscape of the risk groups in TCGA_LUSC using the TIMER, CIBERSORT, XCELL, and three more algorithms in the TIMER2.0 database. The XCELL algorithm revealed significant differences in the infiltration of B cells, monocytes and neutrophils between the two groups (*p* < 0.05, Figure 10). In addition, the TIMER algorithm showed that the infiltration of B cells, neutrophils, myeloid cells and dendritic cells were significantly different between the high-risk and low-risk groups. According to the CIBERSORT algorithm, memory B cells, M1 macrophages and neutrophils showed significant differences in the infiltration rates across the groups (*p* < 0.05, Supplementary Figure 1).

**Figure 11:**
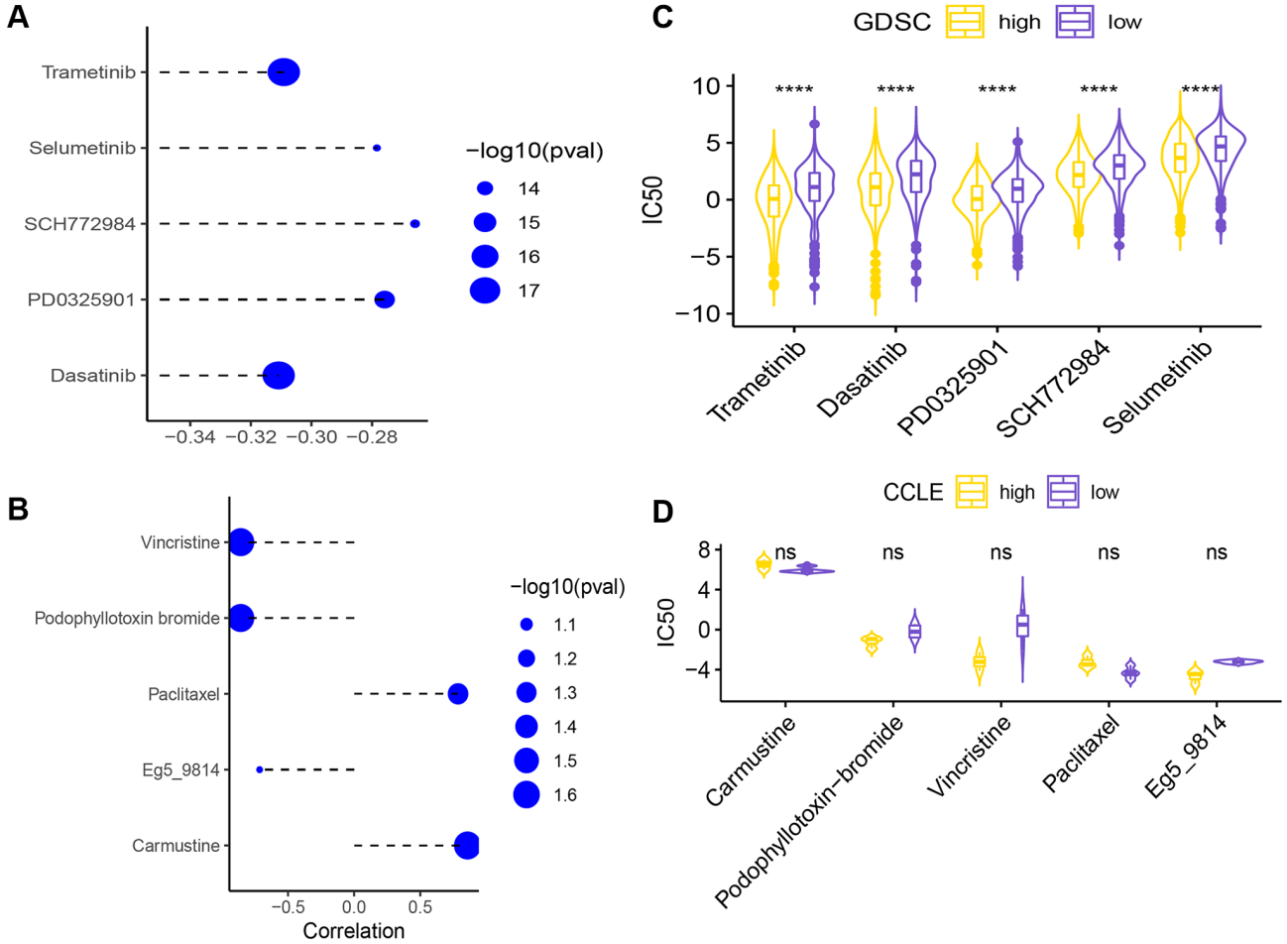
(**A**) Correlation of the necroptosis signature with the sensitivity (IC50) to five drugs. (**B**) Differences in IC50 of 5 drugs between the high-risk and low-risk groups (^****^p < 0.0001). Correlation analysis of signature and 5 drug sensitivity data (IC50) (**C**). The difference analysis of the sensitivity data (IC50) of the five drugs in the high and low groups (**D**) (ns *p* > 0.05).

To explore the differences in chemotherapeutic drug resistance between the two risk groups, we downloaded drug sensitivity data (IC50) and gene expression data from the GDSC database, and used the ssGESA algorithm of the GSVA package to calculate the KEGG pathway enrichment score for each sample. The top 5 drugs with the highest correlation to the necroptosis signature are shown in Figure 11A. The IC50 values of these 5 drugs were significantly different between the high- and low-risk groups (Figure 11B).

Secondly, the drug sensitivity data (IC50) and gene expression data were downloaded from the CCLE database for analysis, and the KEGG necroptosis pathway enrichment score of each sample was calculated using the ssGESA algorithm of the GSVA function in the GSVA package. The high/low groups were distinguished. Here, the top 5 drugs with the smallest spearman correlation *p* value are selected for display (Figure 11C), and the sensitivity data (IC50) of these 5 drugs are not statistically significant between the high and low groups (Figure 11D).

### Validation of the drug sensitivity results *in vitro*

**Figure 12:**
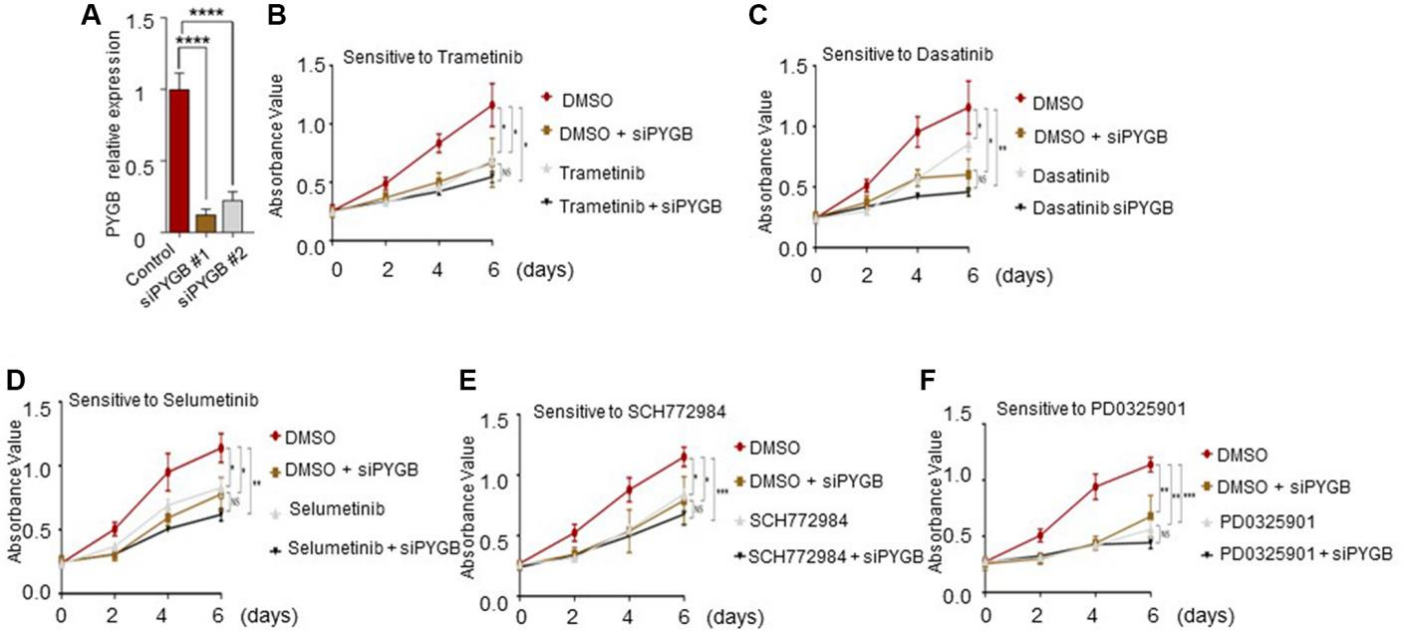
**Effect of the candidate drugs on PYGB-knockdown cells.** (**A**) PYGB expression in the control and siPYGB groups. (**B**–**F**) Viability of the cells in response to (**B**) trametinib, (**C**) dasatinib, (**D**) selumetinib, (**E**) SCH772984 and (**F**) PD0325901.

To validate the candidate drugs identified in the previous section, we knocked down the different necroptosis genes in the A549 cells, and evaluated the viability of respective cell lines in response to the different drugs. As shown in Figure 12, cells with PYGB knockdown showed increased sensitivity to all five drugs compared to the DMSO-treated control cells. Similar results were observed after silencing CHMP4C, JAK1 and IL-1β (Supplementary Figures 2–4). In contrast, cells with TNFRSF10B knockdown were non-viable.

## DISCUSSION

Necroptosis is characterized by loss of cell membrane integrity and passive expulsion of cellular contents [21, 22]. It is regulated by RIPK3, RIPK3 and MLKI [23] and their expression in NSCLC is closely related to prognosis.

A previous study showed that necroptosis led to macrophage-driven adaptive immune suppression in pancreatic ductal adenocarcinoma tumors [7]. In addition, necroptosis plays a crucial role in the malignant transformation of liver cells [24], and mediates the progression of cirrhosis to hepatocellular carcinoma (HCC) [25]. Wu et al. found that necroptosis-related genes including MET, AM25C, MROH9, MYEOV, FAM111B, Y6D, and PPP2R3A are related to the progression of pancreatic adenocarcinoma (PAAD) [6]. Furthermore, tanshinol A (TSA) inhibits the growth of lung cancer cells by triggering necroptosis via MLKL [26]. Therefore, it is crucial to understand the precise molecular mechanisms and signaling events underlying the pro- or anti-tumorigenic role of necroptosis in order to better develop new therapeutic approaches [27].

In our study, we found that the necroptosis pathways were significantly different between the younger (≤60 years) or older (>60 years) subgroups of LUSC, whereas the tumor stage did not show any significant correlation. A previous study reported that old age portended worse prognosis in NSCLC patients [28]. Consistent with our findings, another bioinformatics study on LUSC also did not observe any difference in necroptosis-related pathways between different tumor stages [15, 29]. We analyzed the CNV data of 24 necroptosis genes, of which most showed loss of function. In addition, most necroptosis genes were differentially expressed between the normal lung samples and LUSC samples.

Five necroptosis genes, including CHMP4C, IL1B, JAK1, PYGB and TNFRSF10B, were significantly correlated to the survival. Liu et al. showed that CHMP4C is overexpressed in LUSC patients and portends poor prognosis, and its knockdown induced S-phase arrest in LUSC cells *in vitro* [30]. Furthermore, polymorphisms in the IL1B promoter have been linked to the risk of lung cancer development [31]. Low expression of JAK1 is closely associated with immune infiltration and poor prognosis in lung adenocarcinoma [32]. Likewise, low-level amplification of PYGB epigenetically regulate smoking-induced lung carcinogenesis [33]. High levels of TNFRSF10B mRNA and its encoded protein TRAIL-2 in EGFR wild-type NSCLC are predictive of unfavorable prognosis [34]. Furthermore, the YIPF2-TNFRSF10B axis is closely linked to the malignant progression of NSCLC [35].

The 5-gene necroptosis signature was identified as an independent prognostic biomarker, and was validated in an external dataset. Gao et al. had previously established a prognostic risk score model consisting of 5 genes (MYEOV, LCE3E, PTGIS, OR2W3 and RALGAPA2) for LUSC [29]. In addition, Dai et al. developed a prognostic model for LUSC with six necroptosis-related genes (NRGs), including RIPK3, MLKL TLR2, TLR4, TNFRSF1A and NDRG2. The NRG scores were strongly associated with prognosis, tumor immune microenvironment and tumor mutation burden [15]. We also evaluated the predictive effect of the necroptosis signature on immunotherapy response in other cancer cohorts. While there was no correlation between the signature and survival prognosis in the bladder cancer and renal clear cell carcinoma cohorts, there were significant differences in the response of the high-risk and low-risk bladder cancer, melanoma and renal clear cell carcinoma patients to immunotherapy. Furthermore, the predictive rates of the signature for patient response were respectively 58.6%, 60.8% and 56.1%. Along with tumor-infiltrating immune cell signature, this novel necroptosis signature may help select patients who can benefit the most from anti-PD-1/PD-L1 immunotherapy [36].

The pharmacological data from the Genomics of Drug Sensitivity in Cancer (GDSC) and Cancer Cell Line Encyclopedia (CCLE) are routinely used to identify potential targets of candidate anti-cancer drugs [37], although there are concerns regarding the lack of reproducibility in drug sensitivity measurements across studies [38]. We identified five candidate drugs, including trametinib, selumetinib, SCH772984, PD 325901 and dasatinib, that likely target the necroptosis genes in our model. Furthermore, there were significant differences in the sensitivity of the high-risk and low-risk patients to these drugs, which was also validated through *in vitro* experiments on the A549 cell line.

Trametinib is effective in patients with BRAFV600E-mutant metastatic NSCLC when given in combination with dabrafenib [39, 40]. Furthermore, trametinib has been shown to overcome KRAS-G12V-induced osimertinib resistance in a leptomeningeal carcinomatosis model of EGFR-mutant lung cancer [41]. Selumetinib combined with chemotherapy was associated with a higher response rate in advanced or metastatic KRAS wildtype or unknown non-squamous NSCLC patients [42]. Likewise, the combination of selumetinib and osimertinib was effective in EGFR-mutated NSCLC patients who progressed after EGFR-TKIs [43]. SCH772984 inhibited the proliferation of BRAF or MEK inhibitor-resistant tumor cells by targeting the MAPK signaling pathway [44]. In addition, SCH772984 plus apatinib has been effective against oral squamous cell carcinoma (OSCC) [45], and SCH772984 is also an alternative for the treatment of LKB1 and LKB1/KRAS-mutated NSCLC [46]. Lifirafenib (BGB-283) and mirdametinib (PD-0325901) synergistically inhibited the proliferation of K-RAS-mutated NSCLC cell lines [47]. Dasatinib augmented the effects of anti-PD-1 antibodies in NSCLC models by inhibiting Treg cell transformation and proliferation [48]. Furthermore, dasatinib may be effective against cisplatin-resistant lung cancer by targeting the tumor cells as well as the tumor microenvironment [49].

## CONCLUSION

A necroptosis gene signature was established to predict overall survival and immunotherapy response in LUSC patients. PYGB, CHMP4C, JAK1 and IL-1β may the target genes of trametinib, selumetinib, SCH772984, PD 325901 and dasatinib. Taken together, our study provides new insights into the mechanisms underlying the prognosis of LUSC, which can guide treatment decisions and facilitate personalized treatment. Nevertheless, our study has some limitations. First, we only to validated our findings in a cellular model. Second, we used the A549 cells rather than a squamous lung cancer cell line for the *in vitro* experiments.

## Supplementary Materials

Supplementary Figures

Supplementary Table 1

Supplementary Table 2
