# Supplementary material for: The necroptosis signature and molecular mechanism of lung squamous cell carcinoma
Source: Aging (Albany NY). 2023 Nov 15;15(22):12907–26. doi: 10.18632/aging.205210 (PMC10713426; doi:10.18632/aging.205210)
Supplement: Supplementary Figures [file aging-15-205210-s001.pdf]

SUPPLEMENTARY FIGURES

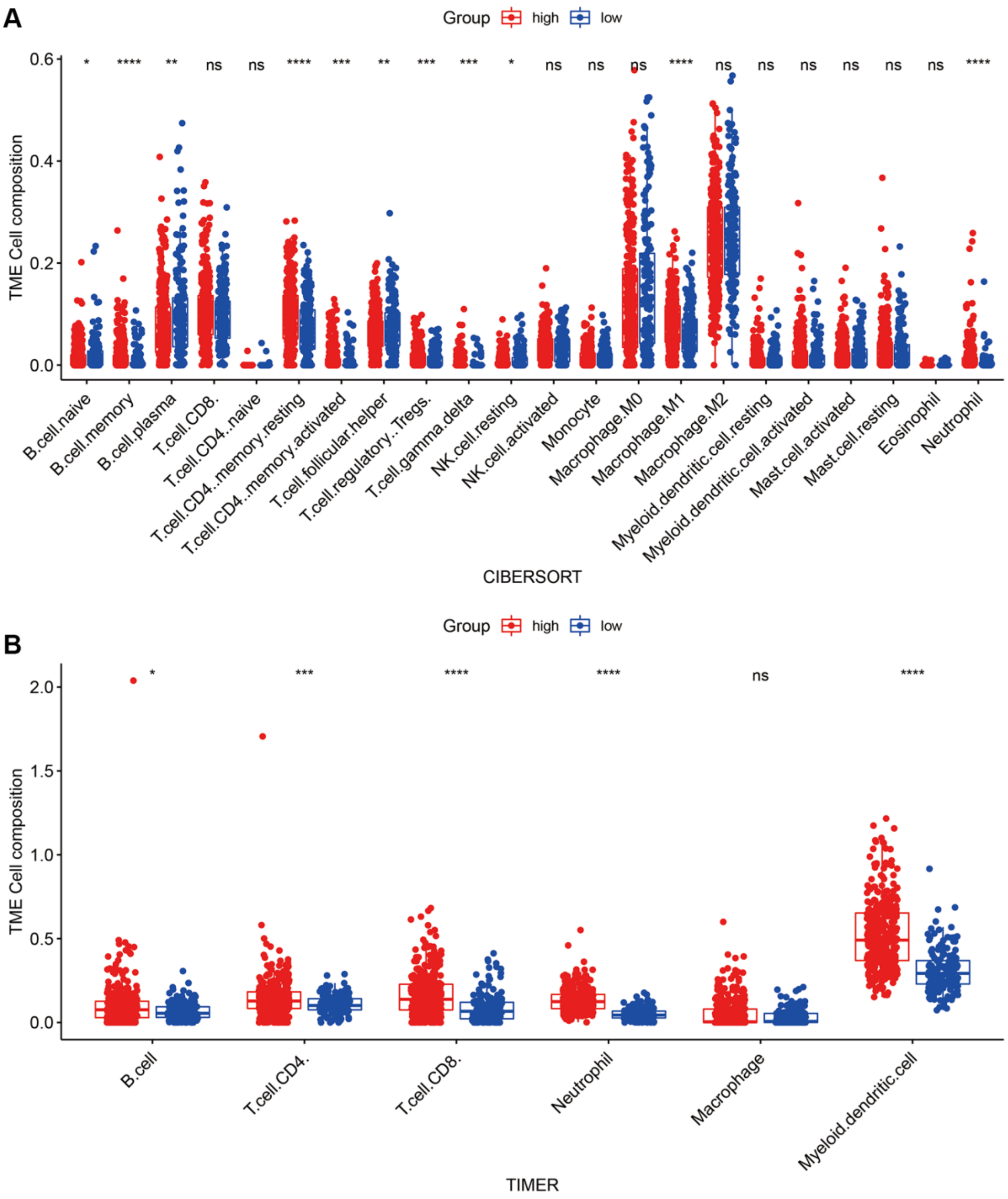

Supplementary Figure 1. The differences in immune cell infiltration between the high-risk and low-risk groups according to the CIBERSORT (**A**) and TIMER algorithms (**B**). ($^{*}p < 0.05$; $^{**}p < 0.01$; $^{***}p < 0.001$; $^{****}p < 0.0001$; ns $p > 0.05$).

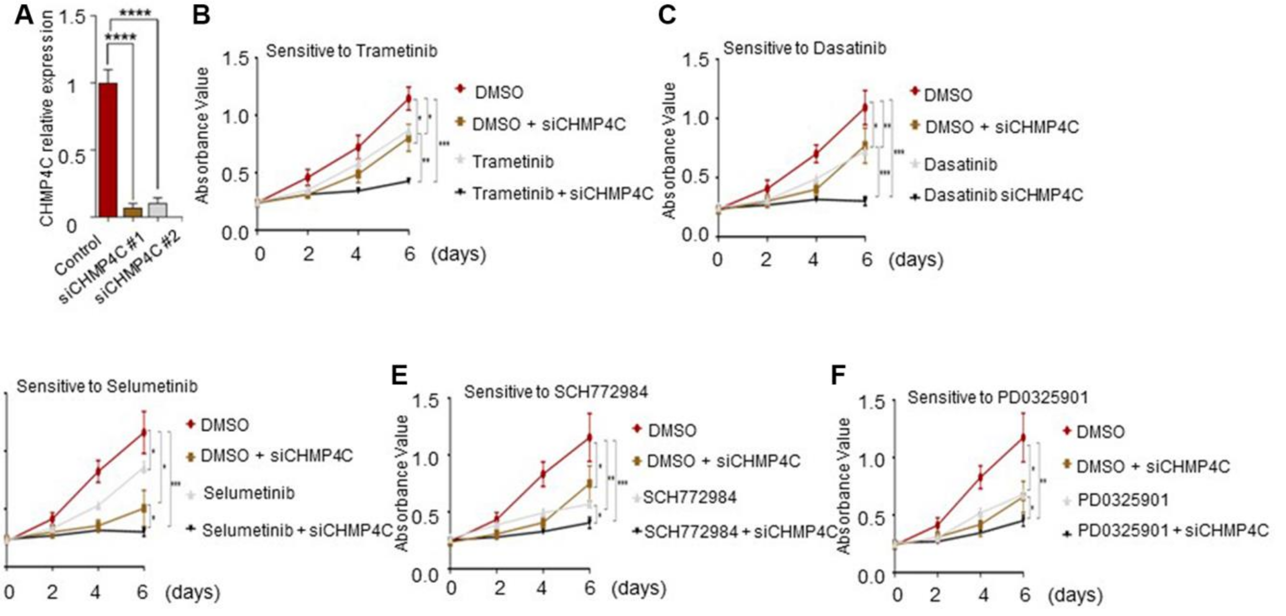

Supplementary Figure 2. Effect of the candidate drugs on CHMP4C knockdown cells. (**A**) CHMP4C expression in the control and siCHMP4C groups. (**B**–**F**) Viability of the cells in response to (**B**) trametinib, (**C**) dasatinib, (**D**) selumetinib, (**E**) SCH772984 and (**F**) PD0325901.

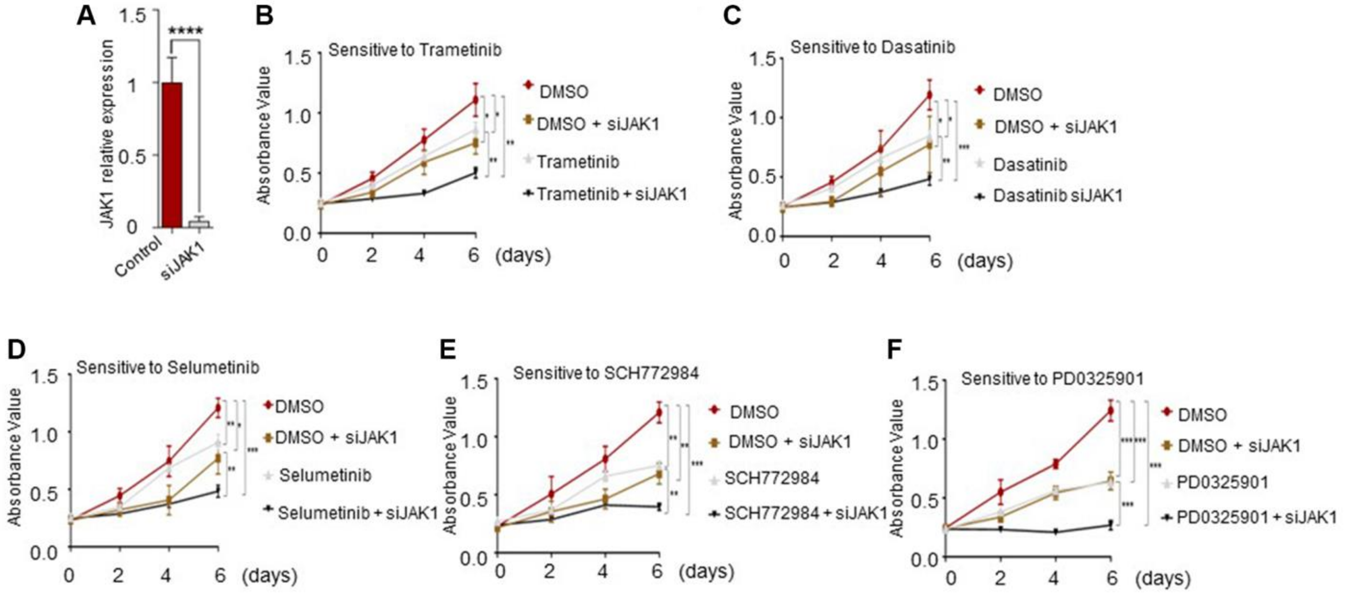

Supplementary Figure 3. Effect of the candidate drugs on JAK1 knockdown cells. (**A**) JAK1 expression in the control and siJAK1 groups. (**B**–**F**) Viability of the cells in response to (**B**) trametinib, (**C**) dasatinib, (**D**) selumetinib, (**E**) SCH772984 and (**F**) PD0325901.

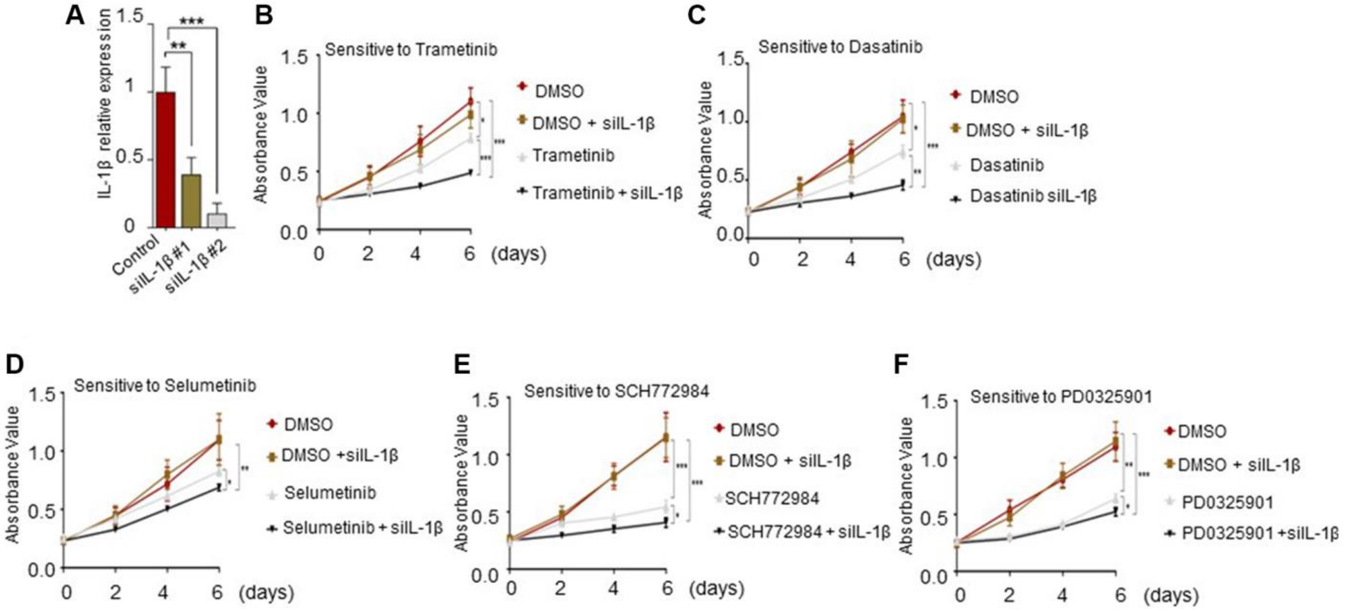

Supplementary Figure 4. Effect of the candidate drugs on IL-β knockdown cells. (**A**) IL-β expression in the control and siIL-β groups. (**B**–**F**) Viability of the cells in response to (**B**) trametinib, (**C**) dasatinib, (**D**) selumetinib, (**E**) SCH772984 and (**F**) PD0325901.
